# Carcinoma of the Breast and Liver

**Published:** 1844-10-05

**Authors:** 


### CARCINOMA OF THE BREAST AND LIVER.

Dr. Fletcher exhibited, at a meeting of the Birmingham Pathological Society, specimens of cancer of the breast and liver, taken from the body of a woman, fifty years of age, who died of the disease. The cancerated breast was ulcerated, and generally carcinomatous; the liver was also carcinomatous, and there were about two gallons of transparent fluid in the cavity of the peritoneum.—*London Medical Times.*

